# I-CONECT: CHALLENGES AND OPPORTUNITIES

**DOI:** 10.1093/geroni/igz038.825

**Published:** 2019-11-08

**Authors:** Hiroko H Dodge, Jeffrey Kaye, Elena Goodrich, Jacob Lindsley, Mattie MacDonald, Nora Mattek, Meysam Asgari, Lisa Silbert

**Affiliations:** 1 University of Michigan, Ann Arbor, Michigan, United States; 2 Oregon Health & Science University, Portland, Oregon, United States

## Abstract

In our previous NIH-funded randomized controlled behavioral clinical trial, we developed a conversation-based social interaction cognitive stimulation protocol delivered by trained interviewers through webcams and a user-friendly interactive Internet interface. Daily 30 minute face-to-face video-chats were conducted for 6 weeks. Despite a short duration, this proof of concept study demonstrated high adherence among older adults (mean age 80 years) and showed improvement in cognitive domains which tap language-based executive functions and semantic memory among the experimental group compared to the control group who did not engage in any video-chats. Building on these results, we are now conducting two NIH-funded projects (https://www.i-conect.org), targeting socially isolated older adults who are less likely to participate in clinical trials despite their high risk of cognitive decline. In this presentation, we introduce a series of projects outlined above and share the challenges and opportunities identified in our behavioral intervention trials focused on social interaction.

